# School Disengagement and Mental Health Service Intensity Need Among Clinically Referred Students Utilizing the interRAI Child and Youth Mental Health Assessment Instrument

**DOI:** 10.3389/fpsyt.2021.690917

**Published:** 2021-12-06

**Authors:** Janell A. Klassen, Shannon L. Stewart, Natalia Lapshina

**Affiliations:** Faculty of Education, Western University, London, ON, Canada

**Keywords:** school disengagement, resource intensity need, referral reason, mental health, interRAI

## Abstract

Although mental health challenges are widespread, impacting 1 in 5 children and youth, only 25% of these young people receive the required mental health supports. Unmet mental health needs are strongly associated with functional impairments including poor self-care, interpersonal challenges, and school difficulties among young people. School disengagement, or a student's lack of involvement in education through interest, curiosity, motivation, and active participation, is associated with a wide array of detrimental outcomes including chronic mental health difficulties, conduct and delinquent behaviors, criminal justice involvement, and unemployment in adolescence and adulthood. Disengagement observed within the school setting may be indicative of underlying mental health challenges and reflective of service intensity need. The current study extends the literature by examining the relationship between school disengagement and mental health service intensity need among 14,750 clinically referred students across elementary and secondary school utilizing the interRAI Child and Youth Mental Health instrument. Findings indicated that more than 25% of clinically referred students were at heighted risk for school disengagement and required high-intensity services. Further, mental health service intensity need was positively associated with risk of school disengagement among students, along with the specific reason for referral (i.e., psychiatric symptoms, harm to self, harm to others, or addiction or dependency), after controlling for sex and age. Implications of the findings are explored within the context of the school setting and future directions are suggested.

## Introduction

An estimated 1.2 million Canadian children and youth experience significant mental illness with clinically significant impairments in functioning requiring treatment (1). Despite a significant number of children and youth demonstrating functional limitations across settings, an alarming number of young people and their families continue to have unmet mental health needs (2, 3). Challenges exist in mental health care for young people across Canada with respect to access to timely and effective treatment as well as coordination of services across sectors [i.e., education, social services, medical, and community-based services; (4, 5)]. Identifying those young people in need of support services and making available the necessary treatments is important to promote positive immediate and life-long outcomes for all Canadians. Certainly, determining the intensity and nature of mental health services required to support a young person and his or her family is a difficult and yet critical step in offering timely and effective treatment opportunities. Although it is widely accepted that mental health challenges are associated with negative educational outcomes, service intensity need has yet to be explored in relation to academic outcomes [e.g., (6)]. Early identification of children and youth in need of mental health services and providing timely access to appropriate treatments is important to promote educational success.

### Mental Health and School Problems

Mental health concerns exhibited by children and youth such as anxiety, attention deficit/hyperactivity disorder, depression, conduct disorder, eating disorders, and suicidal ideation and attempts, are associated with negative educational outcomes (7). A review of the literature on the impact of mental health on school success revealed that “poor academic functioning and inconsistent school attendance are early signs of emerging or existing mental health problems during childhood and adolescence” [(8), p. 189]. Research has consistently demonstrated that mental health challenges can contribute to poor academic achievement, school disengagement, school refusal, and school dropout [e.g., (9–16)].

School problems during childhood and adolescence have been associated with significant negative outcomes. Indeed, early school refusal behaviors, such as school disengagement, increases the risk for later criminal activity, substance use, and school dropout (17). A substantial number of youth involved in the criminal justice system have experienced academic failure, school refusal, school exclusion, and early termination of secondary education (18). A longitudinal study that followed 585 children from age 5 to 27 years old demonstrated that individuals who drop out of secondary school are four times more likely to experience negative outcomes such as being arrested, fired, reliant on government assistance, using illicit substances, and having poor health by 27 years of age (19). Additionally, secondary school dropouts are 24 times more likely to experience as many as four or more of the stated negative outcomes (19). When considering adult outcomes, individuals who dropped out of secondary school make up disproportionately higher percentages of prison inmates as compared to those who completed secondary school (20). Notably, when young people who dropout of secondary school received treatment for behavioral, emotional, or substance use problems before the age 24 years, a reduction in the number of expected negative outcomes has been observed (19). Early identification and timely provision of treatment for children and youth requiring intervention services may reduce the likelihood for the manifestation of acute distress requiring crisis supports both immediately and later in life [e.g., (5, 21)].

### Estimated Value of Services

Significant costs are associated with mental health challenges and delinquency including criminal activity, substance use, and school dropout. Previously, Cohen (22) estimated that typical societal costs for a career criminal, ($1.3–$1.5 million USD), a heavy drug user ($370,000–$970,000 USD), and a high-school dropout ($243,000–$388,000 USD). When taken together, Cohen (22) estimated that the monetary value of saving a high-risk youth was ~$2.3 million USD. Updated estimates of the monetary value of saving a high-risk 14-year-old from a life of negative outcomes range from $2.6 to $5.3 million USD (23). Ultimately, delinquency, including school refusal and school dropout can be both detrimental for individuals and their families as well as expensive for society [e.g., (2, 20)].

Costs associated with supporting children and youth presenting with various mental health challenges has been examined (24). According to the findings, significant discrepancies in expenditures associated with specific diagnoses exist likely because of inconsistent samples and methods for assessing the monetary costs of treatment and the accumulated consequences of unmet treatment needs. Nonetheless, it is clear that when young people do not receive adequate support and treatment, there is an increased likelihood of experiencing significant negative outcomes (i.e., health, mental health, quality of life, unemployment, and poor income), ultimately increasing long-term societal costs (24).

### Service Utilization

Although the first onset of many mental health issues is typically between childhood and early adulthood, children and youth do not always receive the necessary treatment to prevent life-course persistent and chronic mental health problems (2, 25, 26). Research indicates that up to 75% of Canadian children and youth with mental health challenges do not receive required mental health services (2). Early research on patterns of service utilization for addressing mental health challenges among young people indicated that sociodemographic factors, parental attitudes, and the intensity of a child's illness significantly influence service use across settings [i.e., mental health, general health, and school; (27)]. The education system is uniquely situated to identify and support children and youth who are experiencing mental health distress and functional limitations. Not surprisingly, schools were revealed as the main point of entry to mental health services for children and youth (4). The second most common point of entry to mental health services for children up to 13 years old was identified as the specialty mental health sector and for youth 14–16 years old was the juvenile justice system (4). First episode of mental health service utilization among young people tends to “increase in early to middle childhood, stabilize, then increase again in early adolescence” (28). Externalizing behaviors were most predictive of first time service use in middle childhood; however, combined externalizing and internalizing presentation predicted first time service use during adolescence (28).

Parental and adolescent problem recognition are an important step toward service utilization for addressing mental health challenges [for a review see (29)]. Indeed, caregivers play an important role in supporting young people in accessing and participating in mental health interventions (30). Parental beliefs that their child needs help is a critical predictor of service use (27). Parents are more likely to seek services when their child's problems are more severe and persistent, including the presence of comorbidity (29). Additionally, medical issues and school problems were revealed to increase parental help seeking behaviors for young people (29). Consistently, children and youth who acknowledge their experience of psychological distress and related impairments are more likely to seek services (29). Gender differences in help-seeking behaviors were revealed such that males were more likely to access services during childhood and early adolescence, whereas females were more likely to access services in late adolescence (29).

### Current Study

School disengagement, that is a student's lack of meaningful involvement in education as represented by low interest, curiosity, motivation for learning, is associated with varying degrees of challenges for students within the school setting (31). The current study presents a first look at the association between service intensity need and school disengagement among clinically referred students. A strong positive relationship between school disengagement and service intensity need was expected such that students who were disengaged in school were expected to require high-intensity services (i.e., requiring three or more of the following mental health services: inpatient admission; formal care provided by a psychiatrist, psychologist, psychometrist, social worker, child protection worker, or case management; or intervention for life skills training, social skills, crisis management, family functioning, anger management, family preservation, behavior management, family support, and medication management) compared to those students who were engaged in school. Consistent with previously noted age and sex-based findings, it was anticipated that the association between school disengagement and service intensity need at the time of intake to clinical services may differ based on age and sex. Further, primary concerns for referral to mental health services (i.e., psychiatric symptoms, harm to self, harm to others, addiction, or dependency) were investigated to offer insights for triaging purposes.

## Methods

### Participants

Archival *interRAI Child and Youth Mental Health Assessment* [ChYMH; (32)] data collected at seventy community mental health agencies in the Province of Ontario, Canada between November 2012 and May 2019 were utilized for this study. A convenience sample of 14,750 clinically referred young children (*n* = 1,700; ages 4–7 years old), school-aged children (*n* = 4,396; ages 8–11 years old), and youth (*n* = 8,654; ages 12–18 years old) who accessed mental health services was investigated. Participants in this study accessed services through self-referral and referral by healthcare professionals (e.g., family physician or pediatrician), schools, or mental health professionals (e.g., counselor or social worker). The total sample was comprised of English-speaking male (56.2%) and female (43.8%) children and youth ranging in age from 4 to 18 years old (*M*_*age*_= 12.23, *SD*_*age*_= 3.52) who were formally enrolled in schooling (i.e., part-time or full-time). Students were identified as: (a) attending preschool, homeschool, regular classroom with no extra supports; (b) regular classroom with extra support (e.g., classroom, workload, or testing accommodations or modifications such as additional time to complete assessments, oral testing, frequent breaks, withdrawal from class for extra help completing work, one to one support, assistance with personal needs such as feeding or dressing), or; (c) a specialized classroom program (e.g., intellectual, learning, or behavioral needs; vocational training; education within a treatment facility). Refer to Table 1 for more detailed participant characteristics.

**Table 1:** Sample demographic information by age group.

	**Young children**	**School-age children**	**Youth**
	**(*n* = 1,700)**	**(*n* = 4,396)**	**(*n* = 8,654)**
	**(% of subsample)**	**(% of subsample)**	**(% of subsample)**
Age	*M* = 6.20 (SD = 0.91)	*M* = 9.56 (SD = 1.10)	*M* = 14.77 (SD = 1.77)
**Biological sex**			
Male	1,188 (69.9%)	3,039 (69.1%)	4,059 (46.9%)
Female	512 (30.1%)	1,357 (30.9%)	4,595 (53.1%)
**Patient type**			
Inpatient	29 (1.7%)	201 (4.6%)	654 (7.6%)
Outpatient	1,671 (98.3%)	4,195 (95.4%)	8,000 (92.4%)
**Enrollment in school**			
Part-time enrolled	93 (5.5%)	169 (3.8%)	655 (7.6%)
Full-time enrolled	1,607 (94.5%)	4,227 (96.2%)	7,999 (92.4%)
**Education status**			
Pre-school	45 (2.6%)	N/A	N/A
Homeschooled	*	30 (0.7%)	117 (1.4%)
Regular classroom—no extra support	814 (47.9%)	1,621 (36.9%)	4,023 (46.5%)
Regular classroom—extra support	733 (%)	2,103 (%)	2,769 (%)
Specialized school program	101 (%)	642 (%)	1745 (%)
**Reason for referral**			
Specific psychiatric symptoms	822 (48.4%)	2,315 (52.7%)	5,473 (63.2%)
Harm to self	249 (14.6%)	925 (21.0%)	3,050 (35.2%)
Harm to others	557 (32.8%)	1,436 (32.7%)	1,748 (20.2%)
Addiction or dependency	*	16 (0.4%)	702 (8.1%)

* *Ethics approval prohibits reporting on groups smaller than 10 participants*.

### Procedure and Ethical Considerations

Trained assessors (including nurses, psychologists, psychiatrists, social workers, child and youth workers, case managers, and speech and language pathologists) collected data as part of typical clinical practice using a 60–90-min semi-structured interview with the child or youth, caregivers, and collateral contacts (e.g., teachers and therapists) along with any information available with respect to medical and education records. All participants are assigned a case record number upon completion of the assessment tool and no identifying information (e.g., names, full birthday, and postal code) are stored on the interRAI secure server. Data collection using the ChYMH is ongoing across the Province and has been approved by the university ethics review committee.

### Measures

#### The interRAI Child and Youth Mental Health Assessment [ChYMH]

The interRAI ChYMH (32) is a comprehensive assessment tool designed to identify clinically relevant elements pertaining to the specific needs of school-age children and youth (i.e., medical, psychological, social, behavioral, environmental, strengths, and risk). As part of the Child and Youth suite of interRAI assessment tools, instruments within the Child and Youth suite of instruments are being utilized both nationally and internationally. A variety of scales and algorithms are embedded within the instrument to provide tracking indices for measuring symptom severity and to generate data-driven risk assessments across domains (e.g., self-harm, harm to others, and service intensity need). Further, numerous care planning protocols highlighting areas of imminent concern or risk are produced upon completion of the interRAI ChYMH to support clinicians in tracking client progress and in developing adaptive treatment plans. Additional literature with respect to the interRAI assessment can be found on the interRAI website (www.interrai.org). Scales and algorithms developed specifically for the Child and Youth suite of instruments have demonstrated robust psychometric properties including strong inter-rater reliability, internal consistency, as well as substantial face validity, content validity, criterion validity, and discriminant validity [e.g., (33–42)]. Several items, scales and a recently published algorithm from the interRAI ChYMH suite were included in the current research to investigate factors associated with the risk for school disengagement among clinically referred children and youth.

##### School Disengagement

School disengagement among students was evaluated using an eight-item scale, *School Disengagement Scale* (SDeS), including elements of behavioral, emotional, and cognitive disengagement^1^. The presence (0 = *no*, 1 = *yes*) of the items were recorded by assessors (i.e., increased lateness or absenteeism, poor productivity or disruptiveness at school, conflict with school staff, current removal from school due to disruptive behavior, strong persistent dissatisfaction with school, current refusal to attend school, expresses intent to quit school, and poor overall academic performance). The standardized Cronbach's alpha based on the polychoric correlation matrix for the eight items of the SDeS was 0.86, suggesting good reliability. Items were summed and ranged from zero to eight with higher scores indicating an increased risk of school disengagement. Validation research suggests that optimal sensitivity (56.9–76.2%) and specificity (74.1–86.4%) for predicting poor academic performance in the last 6 months is achieved when the cut-off score on the SDeS is two (43**?**). As such, all students in the present study with SDeS scale scores of two or greater were identified as being at risk for school disengagement. Those students with SDeS scale scores of zero or one were identified as being engaged in school.

##### Service Intensity Need

Reflecting the intensity and nature of services required to support children and youth seeking mental health services, the *Resource Intensity for Children and Youth* (RIChY) algorithm was used in this present study (42). The RIChY algorithm is an empirically based decision-support tool composed of 25 individual items, three scales (i.e., *Anxiety, Parenting Strengths, Family Functioning*), and two decision-support algorithms (i.e., *Self-Harm, Harm to Others*) from the ChYMH assessment. Based on critical indicators from the interRAI ChYMH assessment tool, an individual's level of risk is determined using the RIChY to suggest priority for intensive service needs. Variability in critical indicators of service need due to the age of a young person led to the development of three independent but related age-based RIChY decision trees (i.e., 4–7 years old, 8–11 years old, and 12–18 years old). The terminal nodes of the RIChY decision tree range from zero to five, where higher nodes are indicative of higher service intensity need. Strong psychometric properties and clinical applicability have been demonstrated for the RIChY algorithm for its use with children and youth accessing mental health services (42). Notably, children and youth accessing outpatient services scored significantly lower on the RIChY algorithm as compared to children and youth accessing inpatient services (42). Consistent with the published optimal cut-off score for predicting service intensity need, students with RIChY terminal nodes of three or greater were identified as requiring high-intensity services (42). Those students with RIChY terminal nodes of two or less were identified as requiring low-intensity services. Additional information about the RIChY algorithm is available in the identified publication.

### Data Analysis

First descriptive statistics were conducted for all variables using means, standard deviations, and range for the continuous variables and percentage for the categorical variables. Bivariate analyses were conducted to determine the independent associations between school disengagement and service intensity needs with age, gender, and reason for referral. Finally, multivariate binary logistic regression modeling was conducted to examine service intensity need as a function of school disengagement, reason for referral (i.e., psychiatric symptoms, harm to self, harm to others, and addiction), and demographic variables (age and sex) with separate models for each of the investigated age groups (i.e., young children, school-age children, and youth). Notably, addiction as the reason for referral was only computed for youth (age 12–18 years). Variables were considered significant if the *p*-value was <0.05. Odds ratios and 95% CI are reported in **Table 4**. Assumptions testing were conducted for each analysis to control for threats to statistical conclusions.

## Results

### School Disengagement and Service Intensity Need

Findings indicated that 45.9% of students were identified as at risk for school disengagement (young children: 42.1%; school-age children: 47.6%; youth: 45.9%) and 45.5% of students were identified as requiring high-intensity service needs (young children: 23.6%; school-age children: 41.4%; youth: 51.9%) at the time of intake into clinical care. Within this sample, 26.1% of the students (young children: 16.2%; school-age children: 26.3%; youth: 28.0%) were identified as being disengaged in school and as requiring high-intensity service needs. The relationship between school disengagement and service intensity need was examined using separate chi-square analyses for each of the investigated age groups (i.e., young children, school-age children, and youth).

Findings presented in Table 2 revealed that service intensity need was significantly related to school disengagement with low to moderate effects for each of the investigated age groups (i.e., young children, school-age children, and youth). Sex differences in the relationship between service intensity need and school disengagement are also presented in Table 2. As expected, findings indicated that students who require low-intensity services were more likely to also to be engaged in school; conversely, those students who require high-intensity services were more likely to be disengaged in school. Further, sex differences are present in the relationship between school disengagement and service intensity need; however, this relationship is more stable for male students across development as compared to female students.

**Table 2:** Chi-square comparison of service intensity need and risk for school disengagement by sex and age.

	**School disengagement**				**χ^2^ (df)**	***p***	**Cramer's V**	**OR**
	**Engaged**		**Disengaged**					**(95% OR CI)**
	***N***	**(%)**	***N***	**(%)**				
**Young children**								
**Male (*****n*** **=** **1,188)**								
Low service need	521	(84.3)	348	(61.1)	81.62 (1)	<0.001	0.262	3.43
High service need	97	(15.7)	222	(38.9)				(2.60, 4.51)
**Female (*****n*** **=** **512)**								
Low service need	338	(92.1)	92	(63.4)	63.43 (1)	<0.001	0.352	6.71
High service need	29	(7.9)	53	(36.6)				(4.04, 11.16)
**Total (*****n*** **=** **1,700)**								
Low service need	859	(87.2)	440	(61.5)	151.45 (1)	<0.001	0.298	4.26
High service need	126	(12.8)	275	(38.5)				(3.35, 5.42)
**School-age children**								
**Male (*****n*** **=** **3,039)**								
Low service need	1,045	(71.3)	708	(45.0)	215.83 (1)	<0.001	0.266	3.04
High service need	420	(28.7)	866	(55.0)				(2.12, 3.54)
**Female (*****n*** **=** **1,357)**								
Low service need	593	(70.6)	228	(44.1)	94.01 (1)	<0.001	0.263	3.04
High service need	247	(29.4)	289	(55.9)				(2.42, 3.82)
**Total (*****n*** **=** **4,396)**								
Low service need	1,638	(71.1)	936	(44.8)	312.48 (1)	<0.001	0.267	3.03
High service need	667	(28.9)	1,155	(55.2)				(2.68, 3.43)
**Youth**								
**Male (*****n*** **=** **4,059)**								
Low service need	1,227	(64.3)	943	(43.9)	169.37 (1)	<0.001	0.204	2.30
High service need	682	(35.7)	1,207	(56.1)				(2.03, 2.61)
**Female (*****n*** **=** **4,595)**								
Low service need	1,383	(49.8)	606	(33.3)	122.50 (1)	<0.001	0.163	1.99
High service need	1,392	(50.2)	1,214	(66.7)				(1.76, 2.25)
**Total (*****n*** **=** **8,654)**								
Low service need	2,610	(55.7)	1,549	(39.0)	240.19 (1)	<0.001	0.167	1.97
High service need	2,074	(44.3)	2,421	(61.0)				(1.80, 2.14)

### Reason for Referral and School Disengagement

As presented in Table 3, the relationship between reason for referral and school disengagement was examined for each of the investigated age groups (i.e., young children, school-age children, and youth) revealing low to moderate effects. As expected, findings indicated that the specific reason for referral (i.e., psychiatric symptoms, harm to self, harm to others, and addiction or dependency) was uniquely related to the likelihood that students experienced school disengagement.

**Table 3:** Chi-square comparison for school disengagement and reason for referral.

	**School disengagement**				**χ^2^ (df)**	***p***	**Cramer's V**	**OR**
	**Engaged**		**Disengaged**					**(95% OR CI)**
	***N***	**(%)**	***N***	**(%)**				
**Young children**								
Psychiatric symptoms					28.48 (1)	<0.001	0.129	1.69
No	563	(57.2)	315	(44.1)				(1.40, 2.06)
Yes	422	(42.8)	400	(55.9)				
Harm to self					87.38 (1)	<0.001	0.227	3.74
No	908	(92.2)	543	(75.9)				(2.80, 4.99)
Yes	77	(7.8)	172	(24.1)				
Harm to others					144.24 (1)	< .001	.291	3.56
No	777	(78.9)	366	(51.2)				(2.88, 4.40)
Yes	208	(21.1)	349	(48.8)				
**School-age children**								
Psychiatric symptoms					167.32 (1)	<0.001	0.195	2.21
No	1,305	(56.6)	776	(37.1)				(1.96, 2.50)
Yes	1,000	(43.4)	1,315	(62.9)				
Harm to self					128.53 (1)	<0.001	0.171	2.35
No	1,973	(85.6)	1,498	(71.6)				(2.02, 2.73)
Yes	332	(14.4)	593	(28.4)				
Harm to others					299.95 (1)	<0.001	0.261	3.14
No	1,821	(79.0)	1,139	(54.5)				(2.76, 3.59)
Yes	484	(21.0)	952	(45.5)				
**Youth**								
Psychiatric symptoms					134.58 (1)	<0.001	0.125	1.69
No	1,981	(42.3)	1,200	(30.2)				(1.55, 1.85)
Yes	2,703	(57.7)	2,770	(69.8)				
Harm to self					92.36 (1)	<0.001	0.103	1.54
No	3,246	(69.3)	2,358	(59.4)				(1.41, 1.69)
Yes	1,438	(30.7)	1,612	(40.6)				
Harm to others					333.97 (1)	<0.001	0.196	2.72
No	4,078	(87.1)	2,828	(71.2)				(2.44, 3.03)
Yes	606	(12.9)	1,142	(28.8)				
Addiction or dependency					176.13 (1)	<0.001	0.143	2.97 (2.51, 3.51)
No	4,472	(95.5)	3,480	(87.7)				
Yes	212	(4.5)	490	(12.3)				

### Multivariate Analyses

Table 4 presents the results of multivariate binary logistic regression modeling. Each model examined service intensity need as a function of school disengagement, the reason for referral, and demographic variables (age and sex). Separate models examined these relationships for each of the investigated age groups (i.e., young children, school-age children, and youth) and reason for referral (psychiatric symptoms, harm to self, harm to others, and addiction). The model with addiction was computed only for youth (age 12–18 years).

**Table 4:** Multivariate binary logistic regression models for service intensity need as a function of school disengagement, reason for referral, sex and age.

	**Model 1**	**Model 2**	**Model 3**	**Model 4**
	**Psychiatric symptoms**	**Harm to self**	**Harm to others**	**Addiction or dependency**
	**OR (95% OR CI)**	**OR (95% OR CI)**	**OR (95% OR CI)**	**OR (95% OR CI)**
**Age 4–7 years**				
Sex (female vs. male)	0.67 (0.50, 0.89)	0.71 (0.53, 0.94)	0.81 (0.60, 1.09)	
Age	0.89 (0.78, 1.02)	0.89 (0.77, 1.02)	0.88 (0.76, 1.02)	
School disengagement (High vs. Low)	3.99 (3.10, 5.14)	3.61 (2.79, 4.67)	2.99 (2.28, 3.92)	
Reason for referral	1.76 (1.38, 2.23)	3.24 (2.41, 4.36)	6.39 (4.93, 8.28)	
**Age 8–11 years**				
Sex (female vs. male)	1.01 (0.88, 1.16)	1.05 (0.91, 1.20)	1.17 (1.01, 1.35)	
Age	1.09 (1.03, 1.15)	1.07 (1.01, 1.14)	1.11 (1.05, 1.18)	
School disengagement (High vs. Low)	2.78 (2.45, 3.16)	2.79 (2.45, 3.17)	2.43 (2.13, 2.77)	
Reason for referral	1.76 (1.55, 2.00)	2.36 (2.02, 2.76)	3.62 (3.15, 4.16)	
**Age 12–18 years**				
Sex (female vs. male)	1.60 (1.46, 1.75)	1.34 (1.22, 1.47)	2.14 (1.94, 2.35)	1.68 (1.54, 1.84)
Age	1.06 (1.03, 1.08)	1.06 (1.03, 1.08)	1.11 (1.08, 1.14)	1.04 (1.02, 1.07)
School disengagement (High vs. Low)	2.02 (1.85, 2.21)	1.93 (1.75, 2.12)	1.84 (1.68, 2.02)	2.04 (1.87, 2.23)
Reason for referral	1.66 (1.52, 1.82)	5.13 (4.63, 5.68)	4.66 (4.09, 5.31)	2.03 (1.70, 2.41)

As seen in Table 4, in multivariate models, among young children aged 4–7 years, those with high (vs. low) school disengagement and any reason for referral were more likely in odds to require high-intensity services. In particular, young children referred for harm to self were more than three times likely in odds, and those referred for harm to others were more than six times more likely in odds, to require high-intensity services compared to young children with no such referral concerns. In addition, among young children, younger males (compared to females) were more likely in odds to require high-intensity services.

Next, in children aged 8–11 years, those with high (vs. low) school disengagement and any reason for referral were more likely in odds to require high-intensity services. Specifically, school-aged children referred for harm to self were more than two times likely in odds, and those referred for harm to others were more than three times more likely in odds to require high-intensity services compared to school-aged children with no such referral concerns. In this group, females and males had the same likelihood in odds to require high-intensity services. Older children were more likely in odds to require high-intensity services than their younger counterparts within this age range.

Finally, among youth, those with high (vs. low) school disengagement and any reason for referral were more likely in odds to require high-intensity services. In this group, youth referred for harm to self were more than five times more likely in odds, youth referred for harm to others were more than four times more likely in odds, and youth referred for addiction were more than two times more likely in odds to require high-intensity services, compared to youth with no such referral concerns. Notably, in this group, females were more likely in odds to require high-intensity services than males. Older children were more likely in odds to require high-intensity services than younger youth.

## Discussion

Although it is widely accepted that mental health challenges are associated with negative educational outcomes, service intensity need has yet to be explored in relation to school engagement problems among clinical samples of students. The current study contributes to the literature by presenting a first look at the association between school disengagement and service intensity need among clinically referred young children, school-age children, and youth. As predicted, school disengagement was found to be associated with high-intensity service needs. Indeed, students who were at highest risk for school disengagement were ~2–4 times more likely in odds to require high-intensity services. The strength of this relationship differed by age [i.e., young children (4–7 years), school-age children (8–11 years), and youth (12–18 years)] such that young children who were at high risk for school disengagement were more likely to require high-intensity services as compared to their youth counterparts. Further, sex differences indicated that male students who were at high risk for school disengagement were two to three times more likely in odds to require high-intensity services while female students who were at risk for school disengagement were two to seven times more likely in odds to require high-intensity services. The relationship between school disengagement and service intensity need was more stable for male students as compared to female students. Results indicated that young female children who were at heighted risk for school disengagement were found to be almost seven times more likely in odds to require high-intensity services as compared their matched male peers who were only three times more likely in odds to require high-intensity services. Young girls who require high-intensity services is rare, but when this occurs, it is quite significant and highly associated with school disengagement. Interestingly, among school-age children and youth, the likelihood for male and female students to be disengaged in school and require high-intensity services was similar. When investigating service intensity need as a function of school disengagement, reason for referral (i.e., psychiatric symptoms, harm to self, harm to others, and addiction or dependency), and demographic variables (age and sex), similar findings were revealed. Indeed, students of all ages who were identified as being disengaged in school (i.e., as compared to engaged) were more likely in odds to require high-intensity services. Specifically, referral for psychiatric symptoms was associated with two times increased odds for requiring high-intensity services among all students. Further, referral for harm to self was associated with two to five times increased odds for requiring high-intensity services, while referral for harm to others was associated with three to six times increased odds for requiring high-intensity services. Finally, referral for addiction or dependency was associated with two times increased odds for requiring high-intensity services among youth. Findings are considered within the context of the school setting and future directions are suggested.

Research suggests that the severity of presenting concerns is typically associated with the intensity of individualized treatment approaches such that young people who are experiencing more severe distress are more likely to be involved with psychiatric or multidisciplinary supports (3). In this study, students who were at heightened risk for school disengagement, thus experiencing significant challenges within the school setting, were found to be more likely to require high-intensity services. The proportion of students identified as being disengaged in school and requiring high-intensity services increased with age. That is, among clinically referred students, 26% of school-age children and 28% of youth were identified as being disengaged in school and requiring high-intensity services as compared to only 16% of young children. Understandably, young people often rely heavily on their parents for accessing mental health treatment and research suggests that service utilization by children and youth is associated with the health-seeking behaviors of the adults in their household (44). An early study investigating unmet mental health service needs in community samples of children and adolescents revealed that economic disadvantage, parental psychopathology, poor school grades, and parent-reported barriers were key problems for accessing services (45).

It has also been found that parental psychopathology is associated with increased service utilization and expenditures for children and youth, even after controlling for parental service utilization (44, 46). For example, parental depression is associated with increased emergency department use and consultations with general practitioners as well as outpatient and inpatient services by children and youth (44). An investigation of predictors for mental health service utilization among a sample of adolescent males revealed that diagnoses of attention deficit hyperactivity disorder (ADHD) and oppositional defiant disorder (ODD) among adolescent males, as well as parental substance use disorders (i.e., paternal alcohol disorder and maternal amphetamine use disorder) predicted increased mental health service utilization (47). Previous research suggests that young people who acknowledge their distress and related functional impairments are more likely to seek services (29, 45).

Help seeking behaviors associated with mental health services among adolescents and young adults were revealed to be hindered by “perceived stigma and embarrassment, difficulties recognizing symptoms, and a preference for self-reliance” (48). Research consistently indicates that stigma associated with mental illness and mental health treatments can significantly impact an individual's willingness to access and fully participate in treatment services (49). Among 1,092 young Canadians ages 15–24 years old presenting with a mood, anxiety, or substance-related disorders, it was demonstrated that individuals most likely to access mental health services were female, living alone, experiencing challenges in social situations, and presenting with mood disorders or chronic illness (50). Harm to self and others as well as substance use represent forms of maladaptive coping. In the current study, referral for each form of maladaptive coping (i.e., harm to self, harm to others, and addiction or dependency) was found to increase the likelihood for school disengagement for all students.

The education system has been identified as the main point of entry to mental health services for children and youth (4). School staff are uniquely positioned to support students through referrals to more intensive school and community based services. Exploration of the effectiveness of universal screeners as completed by school staff vs. traditional classroom-referral methods for identifying at-risk students revealed that many students requiring mental health support are overlooked when universal screeners are not utilized (51). As indicated in the present study, psychiatric symptoms as well as harm to self and others were related to school engagement problems for all students. Interestingly, although findings revealed that general referrals for psychiatric symptoms increased the likelihood for school disengagement by two to four times across age groups, the likelihood for high service need only increased by about 1.5 times. Consistently, in an investigation of educators' ability to recognize students with mental health concerns within the classroom, teachers were found to be significantly less likely to accurately identify students exhibiting moderate or subclinical mental health symptoms (52). Within the school setting, teachers can consistently detect students exhibiting severe externalizing and internalizing problems (52).

Given the nature of behavioral problems across settings, referral for harm to others was therefore expected to be associated with school engagement problems as well as higher intensity service needs for all students. Findings from the present study revealed strong associations between referral for harm to others, school disengagement, and service intensity need such that students referred for harm to others were between two to three times more likely in odds to be disengaged in school and between four to six times more likely in odds to require high-intensity services compared to students with no such referral concerns. Notably, younger children (ages 4–7 years) who were referred for harm to others were revealed to be at the greatest risk, followed by youth (ages 12–18 years) and finally school-aged children (ages 8–11 years). This is consistent with previous research which indicates that young children are most often referred for externalizing problems such as aggressive and disruptive behaviors whereas youth are referred more for both internalizing and externalizing disorders (28). Further, young children are highly dependent on their caregivers which necessitates significant caregiver involvement in accessing and participating in intervention services. Although the education system is the first most common point of entry to mental health services for children and youth, the second most common point of entry to mental health services for children up to 13 years old is the specialty mental health sector and for youth 14–16 years old was the juvenile justice system (4). In the present study, it may be that older students are just as likely to require high-intensity service needs for harm to others behaviors, however, these students may be involved in services from other sectors (e.g., youth justice) and thus not included in our clinically referred sample.

Results indicated that referral for harm to self was associated with risk of school disengagement and service intensity need. Specifically, students referred for harm to self were between two to three times more likely in odds to be disengaged in school and between two to five times more likely in odds to require high-intensity services compared to students with no such referral concerns. Notably, younger children (ages 4–7 years) who were referred for harm to self were more likely to experience school disengagement meanwhile youth (ages 12–18 years) who were referred for harm to self were more likely to require high-intensity services. Students who are engaging in self-harm require intensive services and support across settings. Within the classroom, self-harm among young students may be more obvious or disruptive in nature as compared to youth who may use adaptive strategies to conceal their self-harming behaviors. As such, school disengagement and self-harm among young students might be more easily detected. Indeed, youth who engage in self-harming behaviors may in fact be high-achieving students with perfectionistic tendencies who are engaged in school, but are struggling with mental health functioning outside of the classroom setting (53, 54). Relatedly, Splett et al. (52) found that teachers rated externalizing behaviors to be more severe and detrimental for the student than internalizing symptoms which may help to explain why self-harm behaviors go unnoticed until the student reaches a point of requiring significant support and intervention.

Of concern particularly among youth, referral for addiction or dependency was found to be associated with an increased likelihood in odds by two times for school disengagement as well as an increased likelihood in odds by two times for requiring high-intensity services as compared to their non-substance addicted counterparts. Although experimentation with risky behaviors such as substance use is common among adolescents, regular substance use can jeopardize an adolescent's physical and mental health and well-being especially given that adolescent substance use is a significant predictor of substance abuse in adulthood (6, 12). Further, substance using teens are at a greater risk for both immediate and long-term consequences such as psychopathology, emotional distress, cognitive impairments, and substance-induced psychosis [e.g., (55, 56)]. Youth who are dependent on substances tend to have significant challenges with managing their drug related behaviors which can interfere with their education. Indeed, directly as related to school outcomes, substance using youth are not able to fully participate in their learning if they are under the influence during school or homework hours. Present findings highlight that drug and addiction education is important among school-age children and youth to reduce the likelihood of addiction and dependency problems which can impact adaptive functioning later in life.

### Summary

Taken together, findings from the current study extend previous research to highlight the relationship between risk of school disengagement and mental health service intensity need among clinically referred students across elementary and secondary school. Indeed, one in four clinically referred students were found to be at risk for school disengagement and requiring high-intensity service needs. School engagement problems within the school setting may be an indicator of underlying mental health problems. School staff are uniquely positioned to support students through early identification and referrals to school and community level supports and services. Significant age and sex differences in the relationship between school disengagement and high-intensity service need suggest the requirement of focused triaging protocols to support students at various stages in development.

### Limitations

Despite the large sample size and use of the interRAI ChYMH, known to be a highly reliable and valid multisource clinician-rated comprehensive assessment tool, the present study should be considered together with its limitations. All participants in the present study were accessing services at a community or inpatient mental health agency, and consequently, generalization of these findings to school-based populations is limited. The examination of school disengagement longitudinally, and prior to referrals to community agencies, would be beneficial to enhance prevention measures to reduce discontinued pursuits to educational attainment. Additionally, racial and cultural information was not obtained and, as a result, examination of these variables in relation to service utilization could not be conducted. Such data will be important to examine to improve social justice, equity as well as the importance of multi-culturally attentive processes and procedures when delivering mental health services.

### Clinical Implications and Future Directions

This research highlights the necessity for early identification and providing timely access to intervention as a method to improve the lives of those at risk for mental health and school problems. Early identification and timely provision of treatment for children and youth requiring mental health services may reduce the likelihood for the manifestation of acute distress requiring crisis supports as well as life-long consequences [e.g., (5, 21)]. Many mental health supports and treatments are provided within the education system; however, the education system is not an appropriate venue to provide all types of treatments required to address psychopathology (e.g., psychiatric intervention, family support, and trauma-focused intervention). Thus, it is critical that sectors involved in supporting children and youth work together in their approach to mental health screening and assessment to foster improved mental health and well-being and to maximize reductions in the negative outcomes that may otherwise be experienced (57). Continuity of care across sectors, namely education, mental health, and medical health services, is essential for ensuring that children and youth demonstrating mental health challenges are provided with appropriate services in a timely manner (4, 24). Implementation of a standardized assessment-to-intervention system within the educational system, the most common point of entry into mental health services, could ultimately improve our mental health delivery system. Such an approach supports early intervention while also facilitating service integration through the use of a common language across service providers, improved triaging and prioritization, and enhanced use of quality data for decision making at a system level^2^. Through the identification of risk and resilience factors, early identification of at-risk students could reduce the likelihood of long-lasting detrimental impacts of school disengagement, resulting in improved outcomes and reducing negative sequalae throughout the lifespan.

## Data Availability Statement

The datasets presented in this article are not readily available to protect the privacy of participants. Requests to access the datasets should be directed to the corresponding author.

## Ethics Statement

Western University's ethics board granted approval for the secondary analysis of data collected in various agencies throughout the Province of Ontario (REB #106415).

## Author Contributions

JA and SS devised the main conceptual ideas for this study, carried out analyses and interpretation of findings, and prepared the manuscript. NL provided data analytical support during revisions for publication. All authors agree to its submission for publication.

## Conflict of Interest

The authors declare that the research was conducted in the absence of any commercial or financial relationships that could be construed as a potential conflict of interest. The Handling Editor JF declared a past co-authorship/collaboration with one of the authors SS.

## Publisher's Note

All claims expressed in this article are solely those of the authors and do not necessarily represent those of their affiliated organizations, or those of the publisher, the editors and the reviewers. Any product that may be evaluated in this article, or claim that may be made by its manufacturer, is not guaranteed or endorsed by the publisher.

## Footnotes

^1^Klassen JA, Hamza CA, Stewart SL. *interRAI's Child and Youth School Disengagement Scale (SDeS): Validation of a New Scale to Measure School Disengagement Among Children and Youth* (manuscript in preparation).

^2^Stewart L, Toohey A. *Screening and assessment of mental health problems in students: utilizing an integrated assessment-to-intervention approach to enhance integrated care*. In: Andrews J, Shaw S, Domene J, McMorris C, eds. *School, Clinical, and Counseling Psychology: Mental Health Assessment, Prevention, and Intervention*. Berlin: Springer (under review).
